# Exploring ADHD understanding and stigma: Insights from an online survey in Lebanon

**DOI:** 10.1371/journal.pone.0310755

**Published:** 2024-11-14

**Authors:** Samar Younes, Aline Hajj, Hala Sacre, Nisreen Mourad, Marwan Akel, Chadia Haddad, Fouad Sakr, Rony M. Zeenny, Pascale Salameh

**Affiliations:** 1 School of Pharmacy, Lebanese International University, Beqaa, Lebanon; 2 INSPECT-LB (Institut National de Santé Publique, d’Épidémiologie Clinique et de Toxicologie-Liban), Beirut, Lebanon; 3 Faculté de Pharmacie, Université Laval, Québec, Canada; 4 Oncology Division, CHU de Québec Université Laval Research Center, Québec, Canada; 5 IVPN-Network, United Arab Emirates; 6 Research Department, Psychiatric Hospital of the Cross, Jall-Eddib, Lebanon; 7 School of Medicine, Lebanese American University, Beirut, Lebanon; 8 School of Pharmacy, Lebanese International University, Beirut, Lebanon; 9 Department of Pharmacy, American University of Beirut Medical Center, Beirut, Lebanon; 10 Faculty of Pharmacy, Lebanese University, Hadath, Lebanon; 11 Department of Primary Care and Population Health, University of Nicosia Medical School, Nicosia, Cyprus; University of Botswana, BOTSWANA

## Abstract

**Background:**

Attention-Deficit/Hyperactivity Disorder (ADHD) is a common neurodevelopmental disorder. Public knowledge of ADHD plays a crucial role in shaping attitudes, reducing stigma, and fostering a supportive environment for individuals with this disease. This study aimed to assess the level of knowledge and stigma of the general Lebanese population regarding ADHD and identify potential factors associated with these variables.

**Methods:**

An online cross-sectional study was conducted between July and August 2023 among adults from all Lebanese regions. The questionnaire was self-administered, available in Arabic and English, and included a sociodemographic section and the validated tools Knowledge of Attention Deficit Disorders Scale (KADDS) and the ADHD Stigma Questionnaire (ASQ).

**Results:**

A total of 647 participants were included. Most participants (n = 483 (74.7%)) lacked prior experience with individuals who have ADHD, and only 12.8% reported having good information about the condition. The participants had a mean knowledge score of 20.49 ±3.23 and a mean ADHD stigma score of 75.71 ±20.58. A significantly higher knowledge score was associated with a university level of education (B = 0.14, p < 0.001), older age (B = 0.14, p = 0.001), using the internet (B = 0.13, p = 0.001) and lecture (B = 0.09, p = 0.015) as a source of ADHD information, having a high monthly income (B = 0.13, p = 0.001), being female (B = 0.08, p = 0.030) and having a health coverage (B = 0.08, p = 0.025). Conversely, a lower knowledge was significantly associated with obtaining ADHD information from television (B = -0.13, p = 0.001) and family (B = -0.08, p = 0.043). A significantly lower score was associated with ever being diagnosed with ADHD (B = -0.18, p < 0.001), having an intermediate monthly income (B = -0.10, p = 0.005), and consuming alcohol (B = -0.11, p = 0.004), while a higher score was significantly associated with a higher KADDS total score (B = 0.12, p = 0.002) and being employed (B = 0.07, p = 0.044).

**Conclusion:**

The present study addressed a gap in the existing literature by examining ADHD knowledge and stigma in the Lebanese population. Surprisingly, a positive correlation between higher ADHD knowledge and increased stigma challenges common assumptions, suggesting a complex relationship between knowledge, misconceptions, and societal attitudes. The findings emphasize the need for targeted education and advocacy to improve knowledge and alleviate misconceptions and stigma within the general population.

## Introduction

Attention-Deficit/Hyperactivity Disorder (ADHD) is the most prevalent abstract behavioral condition, ranking as the second most common chronic illness in children [1]. ADHD is defined as a neurodevelopmental disorder characterized by persistent patterns of inappropriate levels of inattention, hyperactivity, or impulsivity, exerting a significant impact on the personal, social, academic, and occupational functioning and development aspects of affected individuals [2–4]. The various subtypes of attention deficit disorders exhibit distinct prevalence rates within the population afflicted by these disorders [2]. Specifically, the inattentive subtype is observed in approximately 18.3% of the overall patients, whereas the hyperactive/impulsive and combined subtypes account for 8.3% and 70%, respectively [2]. Various studies indicated that the disorders collectively exhibit a ratio of 2:1 in terms of prevalence between males and females [5, 6], where males are prone to exhibiting hyperactive/impulsive symptoms, whereas females are more likely to display inattentive symptoms [7]. ADHD is a phenomenon observed on a global scale, but estimates of its prevalence vary worldwide [4, 8]. Studies have found that ADHD has a worldwide prevalence rate of 5.8% in children and adolescents, while the estimated prevalence in adults is 2.8% [9–11]. Despite extensive research in high-income countries, there is limited data from low and middle-income countries (LMICs) where the majority of the world’s youth live [12]. In Lebanon, a previous ADHD prevalence study reported a prevalence of 3.2% among school children [13].

ADHD is diagnosed clinically through a detailed evaluation of current and previous symptoms and functional impairment, primarily relying on the observation of behavioral symptoms [3, 14]. As per the Diagnostic and Statistical Manual of Mental Disorders fifth edition (DSM-5), ADHD remains a diagnosis that should be considered only after ruling out other mental disorders as potential explanations for the observed behavioral symptoms [15]. The DSM-5 outlines the defining symptoms of ADHD, categorizing them into inattention (11 symptoms) and hyperactivity/impulsivity (9 symptoms) [3, 15]. This classification results in distinct presentations: predominantly inattentive (with 6 or more out of 11 symptoms present), predominantly hyperactive/impulsive (with 6 or more out of 9 symptoms present), combined presentation (fulfilling both criteria), and a partial remission category [3, 15]. For a diagnosis to be confirmed, symptoms of ADHD must be evident in two or more settings before the age of 12, persist for a minimum of 6 months, and result in a notable reduction or impairment in social, academic, or occupational functioning [3, 15]. For adolescents aged 17 and above and adults, a diagnosis requires the presence of at least five symptoms per dimension [3, 15]. Additionally, the International Classification of Diseases (11^th^ revision; ICD-11) differentiates ADHD into five subcategories, mirroring the classifications found in the DSM-5: ADHD combined presentation, ADHD predominantly inattentive presentation, ADHD predominantly hyperactive/impulsive presentation, along with two residual categories, namely ADHD other specified and ADHD non-specified presentation [3]. Unlike the DSM-5, the ICD-11 outlines the fundamental characteristics of the disorder without specifying a precise age of onset, duration, or a specific number of symptoms [16].

The consequences of untreated ADHD for children and their families are substantial where recent studies verified the negative impact of ADHD on the quality of life among children and adolescents [17]. Although the impact on physical functioning is moderate, there is a pronounced impairment in emotional, social, and school functioning which are aggravated by stigmatization hindering the patients’ ability to fully participate in various aspects of life, including education and employment [18, 19]. In fact, the stigma surrounding mental illnesses, including ADHD, is a pervasive challenge [20]. It significantly impacts affected individuals, impeding their inclusion in community activities, health care services, workplaces, and educational institutions [21]. This often leads to feelings of shame, isolation, and reluctance to seek help [22]. Individuals with ADHD may face judgment, skepticism, or even discrimination since they are accused of social norm violation, inducing the disease to themselves, and the sincerity of their symptoms [23].

In Lebanon, ADHD programs encompass various interventions and support services aimed at addressing the needs of individuals with ADHD [24]. These programs include diagnostic services provided by healthcare professionals, treatment options such as medication management and behavioral therapy, educational support through collaborations with schools, and community resources like support groups and advocacy organizations [25, 26]. Despite the presence of these programs, several challenges to care persist. Limited access to resources, social stigma surrounding mental health conditions, cultural beliefs influencing help-seeking behaviors, financial barriers, and a lack of coordination among healthcare providers all contribute to difficulties in accessing and delivering care for ADHD [27]. Addressing these challenges requires a comprehensive approach, including efforts to improve access to resources, increase public awareness and education, reduce stigma, and promote interdisciplinary collaboration among healthcare providers and community stakeholders.

Therefore, understanding the importance of public knowledge of ADHD is crucial, especially when considering the wealth of previously conducted studies that have primarily focused on specific groups, such as parents, healthcare professionals, and teachers [28–35]. Studies have shown that parents with children diagnosed with ADHD often hold numerous misunderstandings about the condition, and many misconceptions about ADHD persist among teachers [32]. In addition, healthcare professionals demonstrated poor to very poor levels of knowledge, perception, and attitude toward ADHD [35]. As for community members, studies reported poor knowledge regarding ADHD etiology and treatment [29].

Public knowledge plays a pivotal role in shaping attitudes, reducing stigma, and fostering a supportive environment for individuals with ADHD. By expanding research efforts to encompass community knowledge, one can gain a more comprehensive understanding of how ADHD is perceived by society. In the Middle East and North Africa (MENA) region, studies have shown insufficient public knowledge of ADHD, but there is paucity of research examining this issue within the Lebanese population [36]. Hence, exploring ADHD in the context of Lebanon, a country with unique cultural and social dynamics, adds to the global discourse on how cultural factors influence the perception and management of ADHD. This knowledge is valuable for researchers and practitioners worldwide who work with diverse populations. Furthermore, this broader perspective can inform targeted educational initiatives and guide policymakers in Lebanon and similar settings to develop evidence-based strategies aimed at enhancing public awareness, ultimately contributing to a more informed community response to ADHD. International stakeholders can adapt these strategies to enhance ADHD care in their regions. Therefore, this study aimed to fill the gap by assessing the level of knowledge and stigma of the general Lebanese population regarding ADHD and identifying potential factors associated with these variables.

## Methods

### Study design

This cross-sectional study was carried out between July 10 and August 26, 2023, across various Lebanese regions. It was conducted using an online questionnaire created on Google Forms, and the weblink to the questionnaire was shared on Facebook, LinkedIn, and WhatsApp. The online questionnaire made it easy to reach urban and remote areas, therefore facilitating broad geographic reach and convenience for participants. The sample was purposefully selected through convenient sampling from the five governorates of Lebanon (Beirut, Beqaa, Mount Lebanon, South, and North), and a snowball technique was applied to include other participants. At the beginning of the questionnaire, a cover letter was included that briefed the respondents about the topic and the different aspects of the questionnaire before filling it out while being assured of the anonymity of their responses. Participants were informed that their involvement was voluntary, and they were guaranteed that their answers would be kept confidential.

All Lebanese people aged 18 years and above and living in Lebanon were eligible to participate. Participation was voluntary, and respondents were not incentivized to join the study.

### Ethical aspect

The Lebanese International University School of Pharmacy Research and Ethics committee approved the study protocol (2023RC-020-LIUSOP). Written consent was obtained from each person at the beginning of the questionnaire.

### Sample size calculation

The minimum sample size was calculated using the CDC Epi-info software. The expected frequency was kept at 50% to yield the largest minimal sample size. Accordingly, a minimal sample size of 384 participants was required to produce a 95% Confidence Interval, with a 5% alpha error and a power of 80%.

### Questionnaire

The questionnaire was in Arabic and English, thus allowing people to complete it in their preferred language (S1 Appendix). These languages were chosen because Arabic is the mother tongue of all respondents, while English is commonly used in the country. The questionnaire included a sociodemographic section about the family and a scale-based category comprising validated ADHD knowledge and stigma scales.

#### Sociodemographic data

In this part of the questionnaire, participants were asked about their general sociodemographic data, including age, gender, nationality, area of residence, educational level (primary education including kindergarten through fifth or sixth grade, intermediate education including sixth through eighth grade, and secondary education including ninth through twelfth grade; as well as university education), income, marital status, smoking (including cigarettes, electronic cigarettes, cigars, and waterpipe), and alcohol consumption, health status and diseases, having a health professional member in the family, healthcare access, number of children/dependents and information about house crowding index. This first part also included a question exploring if the participant knew anyone having ADHD or had information about the disease.

#### Scale-based category

The following scales were used in questionnaire:

*Knowledge of attention deficit disorders scale (KADDS)*. This 36-item instrument measures the level of knowledge regarding ADHD related to three subdomains, i.e., associated features (15 questions relating to general aspects of ADHD as prevalence, risk factors, and causes); symptoms/diagnosis (9 questions relating to signs and symptoms of attention deficit, hyperactivity, behavior, and associated mood conditions); and treatment (12 questions relating to nonpharmacological therapy, including behavioral, lifestyle, and electroconvulsive therapy; as well as available pharmacological options with their possible adverse events). All items have three options for answers (yes, no, and I don’t know). Total scores were calculated based on the percentage of items answered correctly [27, 37, 38]. All the KADDS questions with highlighted correct answers are available in S1 Appendix. In its original version, the KADDS scale reported a Cronbach’s alpha of 0.71 for each subscale and 0.86 for the general scale; thus, it has an acceptable internal consistency [27]. In this study, the Cronbach’s alpha was 0.954.

*The ADHD Stigma Questionnaire (ASQ)*. The ASQ is a 26-item self-reported measure of ADHD-related stigma across three factors: disclosure concerns, negative self-image, and concern with public attitudes. Items are rated on a 5-point Likert scale from “strongly agree” to “strongly disagree”, with higher overall stigma scores indicating more stigmatization beliefs [39] (Cronbach’s alpha  =  0.974).

### Translation procedure

A senior research member, who is a medical editor, evaluated and verified the translation (from English to Arabic) performed by one of the collaborators while developing the questionnaire. Minor discrepancies were resolved by consensus among all the collaborators. The questionnaire was piloted among 10 individuals to ensure questions’ clarity. Notably, the final questionnaire was in both Arabic, the native language of Lebanon, and English, the original language of the scales that is commonly understood in Lebanon.

### Statistical analysis

The collected data were analyzed using Statistical Package for Social Sciences (SPSS) version 28.0. For descriptive analysis, frequencies and percentages were used for categorical variables, and means and standard deviations were used for quantitative variables. For dependent variables, the median and interquartile ranges were presented; the distribution of these variables was deemed normal upon visual inspection of the histogram, while the skewness and kurtosis were lower than 1. These conditions are considered compatible with normality with a sample size higher than 300.

For the bivariate analysis of continuous variables, the Student’s T-test was used to compare the means between two groups, and ANOVA was applied to compare between three groups or more after checking for homogeneity of variances using Levene’s test. The corrected T-test and the Kruskal-Wallis test were used when the variances were not homogenous. After ANOVA and Kruskal-Wallis significant testing, post hoc analyses were conducted using the Bonferroni adjustment. A Pearson’s correlation coefficient was used between continuous variables. In all cases, a p-value lower than 0.05 was considered significant.

As for the multivariable analysis, two linear regressions were conducted taking the knowledge and stigma toward ADHD as the dependent variables (outcome variables). The normality of the variables was checked by the residues’ normality, the linearity of the relationship, the absence of multicollinearity, and homoscedasticity assumptions. Also, the Cook’s distance was performed to find influential outliers among the predictor variables. A stepwise method was used to reach the most parsimonious model. The beta coefficient, its 95% Confidence Interval, and the p-value were reported. Independent variables introduced in the models were those that had a p-value lower than 0.2 in the bivariate analysis, considering the maximum number allowed of variables to be included given the sample size: sociodemographic and other independent variables were added as appropriate.

## Results

### Sociodemographic characteristics

A total sample size of 647 participants was reached. The majority of participants were females (63.2%), single/widowed/divorced (74.7%), unemployed (64.8%), non-alcoholics (78.5%), non-smokers (77%), had a university-level education (88.7%), and health insurance coverage (63.7%). The mean age of the participants was 26.52 ± 9.14 years, and 37.1% were students. Table 1 shows the detailed sociodemographic characteristics of participants.

**Table 1:** Sociodemographic characteristics of the participants (N = 647).

Variables	Mean ± SD
**Age**	26.52 ± 9.14
	**N (%)**
**Gender**	
Male	238 (36.8%)
Female	409 (63.2%)
**Marital status**	
Single/widowed/divorced	483 (74.7%)
Married	164 (25.3%)
**Education level**	
Primary	9 (1.4%)
Intermediate	16 (2.5%)
Secondary	48 (7.4%)
University	574 (88.7%)
**Monthly income**	
Don’t know/prefer not to answer	242 (37.4%)
Low (< 100 USD)	114 (17.6%)
Intermediate (100–335 USD)	152 (23.5%)
High (> 335 USD)	139 (21.5%)
**Area of residence**	
Beirut	145 (22.4%)
Mount Lebanon	136 (21.0%)
North	108 (16.7%)
South	107 (16.5%)
Beqaa	151 (23.3%)
**Occupation**	
Employed	228 (35.2%)
Unemployed	419 (64.8%)
**Field of employment**	
Health related field	70 (30.7%)
Non health related field	158 (69.3%)
**Alcohol consumption**	
Yes	139 (21.5%)
No	508 (78.5%)
**Smoking cigarettes**	
No	498 (77.0%)
Yes, previous smoker	44 (6.8%)
Yes, current smoker	105 (16.2%)
**Smoking waterpipe**	
No	398 (61.5%)
Yes, previous smoker	59 (9.1%)
Yes, current smoker	190 (29.4%)
**Smoking e-cigarettes/vaping devices**	
No	518 (80.1%)
Yes, previous smoker	60 (9.3%)
Yes, current smoker	69 (10.7%)
**Health coverage**	
Yes	412 (63.7%)
No	235 (36.3%)

### Participants’ experiences with ADHD

Most participants (n = 483, 74.7%) lacked prior experience with individuals who have ADHD, and 62.3% had no close personal relationships with individuals affected by ADHD. Furthermore, only around 5% of the participants had ever been diagnosed with ADHD. Regarding knowledge of ADHD, only 12.8% reported having good information about the condition, while 12.7% had attended conferences or seminars related to ADHD, and 9.1% had received full professional training on how to interact with individuals with ADHD. The primary sources of information about ADHD were the internet (68.9%) and social media (63.7%). Table 2 reports the participants’ full experiences with ADHD.

**Table 2:** Description of participants’ experiences with ADHD (N = 647).

Variable	N (%)
**Experience with individuals with ADHD**	
Yes	164 (25.3%)
No	483 (74.7%)
**Closest approximation of relationship with individuals with ADHD**	
No relationship	403 (62.3%)
Immediate family (father, mother, brother, sister, son, daughter)	64 (9.9%)
Extended family (uncle, aunt, grandfather, grandmother, grandson, granddaughter)	41 (6.3%)
Friend or acquaintance	64 (9.9%)
Patient of mine	11 (1.7%)
Any other	64 (9.9%)
**Ever been diagnosed with ADHD**	
Yes	32 (4.9%)
No	615 (95.1%)
**Knowledge of ADHD**	
I don’t know anything about it	175 (27.0%)
I have some information about it	389 (60.1%)
I have good information about it	83 (12.8%)
**Ever attended a conference or seminar on ADHD**	
Yes	82 (12.7%)
No	565 (87.3%)
**Received full professional training to interact with individuals with ADHD**	
Yes	59 (9.1%)
No	588 (90.9%)
**Sources of information about ADHD**	
Family	152 (23.5%)
Friends	199 (30.8%)
Books/journals/newspapers/magazines	247 (38.2%)
Internet	446 (68.9%)
Social media	412 (63.7%)
Television	207 (32.0%)
Class lectures	236 (36.5%)
Webinars, seminars, conferences	150 (23.2%)

### ADHD knowledge and stigma

The participants had a mean KADDS score of 20.49 ±3.23. Breakdowns of the KADDS subscale scores revealed mean scores of 8.03 ±1.65 for associated features, 5.53 ±1.92 for symptoms/diagnosis, and 6.92 ±1.56 for treatment. On the other hand, the mean score of ASQ was 75.71 ±20.58. When standardized, these values are considered average. The mean, median, minimum, and maximum values for both the KADDS scale and its subscales and ASQ are shown in the S1 Table.

### Bivariate analysis

In the bivariate analysis, taking the KADDS score as the dependent variable, females had significantly higher scores compared to males (P = 0.018). Additionally, participants with a university-level education showed significantly higher scores than those with a school-level education (P < 0.001), as did individuals with health coverage compared to those without (P = 0.003). Furthermore, participants who works in the health field (p = 0.047) and who reported prior experience with individuals affected by ADHD (p = 0.017) had significantly higher scores in comparison to those who do not work on health field and do not have such experience. Participants who sourced their ADHD information from books, journals, newspapers, or magazines also scored significantly higher than those who did not (P = 0.028). Moreover, participants who obtained ADHD-related information from the internet exhibited significantly higher scores (P < 0.001), as did those who received information through class lectures (P = 0.032). Significant variations in KADDS scores were observed among different categories of monthly income (P < 0.001) (S2 Table). Lastly, there was a significant correlation between participants’ age and their KADDS scores; older participants had better ADHD knowledge than younger participants (less than 30 years) (r = 0.102, P = 0.009).

In the bivariate analysis, taking the ADHD Stigma Questionnaire score as the dependent variable, being employed vs non (p = 0.022), being a non-alcohol consumers vs those who consume alcohol (P = 0.001) exhibited significantly higher scores of stigma scale. Additionally, participants who had never been diagnosed with ADHD had significantly higher scores than those who had received such a diagnosis (P < 0.001). Furthermore, participants who had not received training to interact with individuals affected by ADHD had significantly higher stigma scores compared to those who had received such training (P < 0.001). Participants who did not receive ADHD-related information from their family members also had significantly higher scores compared to those who did (P = 0.011). Moreover, individuals who sourced their ADHD information from the internet scored significantly higher compared to those who did not (P = 0.025). A significant difference in the stigma score was also observed among various categories of monthly income (P = 0.001) (S2 Table). Lastly, there was a significant correlation between the KADDS total score and the ADHD Stigma Questionnaire score (r = 0.142, P < 0.001). Table 3 shows the bivariate analysis, taking the KADDS and ASQ scores as the dependent variables.

**Table 3:** Bivariate associations of the KADDS and ASQ scores with the sociodemographic characteristics.

		Total N	Knowledge of Attention Deficit Disorders Scale (KADDS)	t-statistic	p-value*	Attention-Deficit/Hyperactivity Disorder (ADHD) Stigma Questionnaire	t-statistic	p-value*
**Gender**								
Male		238	20.11±2.99	**-2.37**	**0.018**	75.01±19.61	-0.66	0.511
Female		409	20.71±3.34			76.11±21.14		
**Marital status**								
Single/widowed/divorced		483	20.39±3.22	-1.30	0.192	75.69±20.32	-0.02	0.981
Married		164	20.78±3.25			75.74±21.39		
**Education level**								
School level		73	19.08±3.25	-4.01	**<0.001**	72.91±19.94	-1.23	0.219
University level		574	20.67±3.18			76.06±20.65		
**Occupation**								
Employed		228	20.69±3.28	-1.14	0.253	78.22±19.81	-2.30	**0.022**
Unemployed		419	20.38±3.20			74.34±20.88		
**Field of employment**								
Health		70	21.34±3.74	2.00	**0.047**	77.61±21.63	-0.31	0.756
Non health		158	20.40±3.02			78.50±19.02		
**Health coverage**								
Yes		412	20.77±3.30	-2.94	**0.003**	74.71±20.87	0.08	0.102
No		235	20.00±3.04			77.46±19.99		
**Alcohol consumption**								
Yes		139	20.73±3.36	-0.98	0.328	70.20±21.89	3.41	**0.001**
No		508	20.43±3.19			77.21±19.97		
**Smoking cigarettes**								
Yes		105	20.46±3.15	0.10	0.919	74.52±23.59	0.58	0.565
No		542	20.50±3.24			75.94±19.96		
**Smoking nargileh**								
Yes		190	20.30±2.98	0.97	0.333	76.58±21.94	-0.69	0.487
No		457	20.57±3.32			75.34±20.01		
**Smoking e-cigarettes/vaping devices**								
Yes		69	19.81±3.01	1.86	0.063	72.59±22.59	1.33	0.184
No		578	20.57±3.24			76.08±20.32		
**Ever been diagnosed with ADHD**								
Yes		32	19.53±3.01	1.73	0.083	54.90±16.43	6.02	**<0.001**
No		615	20.54±3.23			76.79±20.21		
**Experience with individuals with ADHD**								
Yes		164	21.22±3.26	-2.41	**0.017**	76.43±21.79	-0.36	0.716
No		353	20.47±3.32			75.72±20.09		
**Been trained to interact with individuals with ADHD**								
Yes		59	20.35±3.64	0.35	0.727	66.40±22.00	0.37	**<0.001**
No		588	20.51±3.19			76.64±20.22		
**Sources of information about ADHD**								
Family	Yes	152	20.07±3.35	1.82	0.069	71.65±23.12	2.55	**0.011**
	No	495	20.62±3.18			76.95±19.59		
Books	Yes	247	20.85±3.41	-2.20	**0.028**	74.03±22.40	1.58	0.116
	No	400	20.27±3.09			76.74±19.33		
Internet	Yes	446	20.78±3.34	-3.61	**<0.001**	76.92±20.81	-2.24	**0.025**
	No	201	19.85±2.87			73.01±19.86		
Social media	Yes	412	20.63±3.43	-1.48	0.138	75.92±21.80	-0.36	0.717
	No	235	20.25±2.82			75.34±18.29		
Television	Yes	207	20.16±3.34	1.79	0.073	76.40±21.40	-0.58	0.559
	No	440	20.65±3.17			75.38±20.20		
Class lectures	Yes	236	20.85±3.42	-2.15	**0.032**	73.63±22.34	1.87	0.061
	No	411	20.28±3.10			76.90±19.43		

*Number in bold are statistically significant (< 0.05).

### Multivariable analysis

Two multivariable linear regression models were conducted to examine factors associated with ADHD knowledge and stigma. The normality of the scales was checked by the P-p plot for the two-regression analysis taking the knowledge and stigma of ADHD as the dependent variables. The results showed that the points on the figures follow approximately along the diagonal line concluding that the data is approximately normally distributed (S1 Fig). S2 Fig demonstrated the Cook’s distance values of the two-regression analysis taking the knowledge and stigma toward ADHD as the dependent variables to find influential outliers. In this particular dataset there were 647 observations, so any observation with a Cook’s distance greater than 4/647 = 0.006 were considered to be highly influential. In the first figure (S2A Fig) almost all the points are under 0.006 accordingly the observations are well represented. On the second figure (S2B Fig) some points are above the threshold indicating an influential observation.

The first model considered the KADDS total score as the dependent variable. A significant regression was found F (9, 637) = 9.062, p<0.001 with an R^2^ of 0.101. A significantly higher KADDS score was associated with a university level of education (B = 0.14, p < 0.001), older age (B = 0.14, p = 0.001), using the internet (B = 0.13, p = 0.001) and lecture (B = 0.09, p = 0.015) as a source of ADHD information, having a high monthly income (B = 0.13, p = 0.001), being female (B = 0.08, p = 0.030) and having a health coverage (B = 0.08, p = 0.025). Conversely, a lower KADDS score was significantly associated with obtaining ADHD information from television (B = -0.13, p = 0.001) and family (B = -0.08, p = 0.043) (Table 4, Model 1).

**Table 4:** Multivariable linear regression taking the knowledge and stigma toward ADHD as the dependent variables.

**Model 1 considering the Knowledge of ADHD scale as the dependent variable**			
	**Standardized Beta**	**t-statistic**	**p-value**
Education level (University vs. school*)	0.14	3.57	<0.001
Age	0.14	3.53	<0.001
Sources of information about ADHD—Internet (Yes vs. No*)	0.13	3.22	0.001
Monthly income (High vs. do not know*)	0.13	3.44	0.001
Sources of information about ADHD—TV (Yes vs. No*)	-0.13	-3.25	0.001
Gender (Female vs. male*)	0.08	2.17	0.030
Health coverage (Yes vs. No*)	0.08	2.24	0.025
Sources of information about ADHD—lecture (Yes vs. No*)	0.09	2.44	0.015
Sources of information about ADHD—family (Yes vs. No*)	-0.08	-2.02	0.043
Adjusted R^2^ = 0.101; F-value = 9.062; df = 637, p<0.001			
**Model 2 considering the stigma of ADHD scale as the dependent variable**			
Ever been diagnosed with ADHD (Yes vs. No*)	-0.18	-4.78	<0.001
KADDS knowledge scale	0.12	3.11	0.002
Monthly income (Intermediate vs. do not know*)	-0.10	-2.81	0.005
Alcohol (Yes vs. No*)	-0.11	-2.91	0.004
Occupation (Employed vs unemployed*)	0.07	2.01	0.044
Adjusted R^2^ = 0.089; F-value = 13.58; df = 641, p<0.001			

Variables entered in model 1: Gender, age, education level, marital status, health coverage, smoking vape, have an experience with individuals with ADHD, Source of information, household income and field of occupation.

Variables entered in model 2: age, occupation, monthly income, health coverage, smoking vape, alcohol use, ever been diagnosed with ADHD, trained to interact with individuals with ADHD, source of information and KADDS knowledge scale.

*Reference group

The second model considered the ASQ score as the dependent variable. A significant regression was found F (5, 641) = 13.58, p<0.001 with an R^2^ of 0.089. A significantly lower score was associated with ever being diagnosed with ADHD (B = -0.18, p < 0.001), having an intermediate monthly income (B = -0.10, p = 0.005), and consuming alcohol (B = -0.11, p = 0.004), while a higher score was significantly associated with a higher KADDS total score (B = 0.12, p = 0.002) and being employed (B = 0.07, p = 0.044) (Table 4 and Model 2).

## Discussion

This study examined the knowledge of ADHD and stigma among a sample of the general Lebanese population, adding to the scarce body of knowledge. Indeed, the available literature mainly focuses on knowledge and stigma in specific populations, such as teachers, parents of children with ADHD, and adolescents with or at high risk for developing ADHD, but very few addressed this topic in the general population.

The findings of the study showed that almost three-quarters of the participants lacked prior experience with individuals diagnosed with ADHD, and more than half of them reported having no close personal relationships with people affected by ADHD, highlighting the potential for misconceptions and stigma due to the limited direct exposure and personal contact with people who have ADHD.

The knowledge related to ADHD, as measured by the KADDS scale, was average (just above 50%). Only 12.8% reported having good information about the condition, while the majority used the internet and social media as primary sources of information about ADHD. A mere 32% sought information from television. Notably, sourcing information from the internet and lectures were associated with significantly better overall knowledge of ADHD, while relying on television and family to learn about ADHD was linked to lower knowledge. While several studies have shown a preference towards the internet and social media as sources of information, knowledge scores differed from one study to another and one country to another. In line with our findings, a study from Saudi Arabia reported low knowledge (10.3%) of ADHD in the general population, primarily derived from social media, although it did not use a validated tool to measure knowledge [36]. In the United States, a study comparing ADHD knowledge of two samples of adolescents (high versus low risk for developing ADHD) and their parents revealed higher knowledge levels, likely due to self-rating, but also revealed notable misperceptions in both cohorts [29]. The preference for sourcing information from social media was evident. Consequently, given the prevailing trend of relying on the internet for health information, educational efforts should underscore the importance of verifying the accuracy and reliability of internet-based health information [29]. Regarding television as a source of information, a study indicated that parents seeking information from TV demonstrated relatively good knowledge about symptoms of ADHD but lacked substantial understanding of its diagnosis, treatment, and prognosis and held incorrect beliefs [31]. This finding suggests that TV may not provide comprehensive and accurate information about ADHD. Thus, strategies, such as delivering thorough ADHD education through television programs involving medical professionals to ensure information reliability, could be implemented to mitigate this impact. Additionally, promoting alternative reliable sources, such as reputable websites, books, and educational programs, may enhance the knowledge of individuals seeking information on ADHD through television. Moreover, the current findings indicate that reliance on lectures was associated with better knowledge about ADHD, while information from family members was linked to lower knowledge levels. This may be attributed to the structured and evidence-based nature of lectures, which often provide comprehensive and up-to-date information delivered by experts in the field [32]. In contrast, information from family members may lack accuracy and depth, potentially leading to misconceptions or incomplete understanding of ADHD. These results align with previous research highlighting the importance of formal educational sources in acquiring accurate knowledge about medical conditions, as opposed to informal, less reliable sources [29].

Consistent with previous studies of mental health and ADHD knowledge correlates [32, 36, 40], female gender, higher educational level, and higher and consistent socioeconomic and work statuses were significantly associated with better ADHD literacy and knowledge, highlighting that public mental health education may have disparate effects on individuals based on demographic characteristics and social standing. In this study, age was found to be a predictor of higher knowledge levels, contrary to some published papers [32, 36, 41]. However, a cautious interpretation is necessary, given our participant group’s relatively young age (mean age: 26.52 years) with high educational levels (89% at the university level).

Surprisingly, our study revealed a positive correlation between a higher knowledge of ADHD and an increased incidence of stigma. In line with this, a previously conducted study among school teachers showed that, despite having poor knowledge and significant misconceptions regarding ADHD, participants exhibited a positive attitude [42]. However, our study finding contradicts most of the existing research showing that the general lack of knowledge associated with ADHD might lead to myths and stigmatization that adversely affect individuals dealing with the condition [43, 44]. Despite this apparent paradox, several hypotheses can be considered to explain the observed result. Stigma often arises from negative stereotypes or perceptions rooted in misinformation or misunderstandings about ADHD, which may not necessarily align with knowledge of the condition. An information bias could also be incriminated in such a finding, related to the KADDS tool specificity and the self-reported nature of the scale items. Notably, only 13% of our participants reported possessing accurate information about ADHD, suggesting a potential overestimation of knowledge or the presence of misconceptions. For instance, some participants may hold the belief that ADHD lacks legitimacy as a genuine medical condition or subscribe to the notion that individuals with ADHD only need to exert more effort [45]. Such attitudes contribute significantly to the perpetuation of stigma [45, 46]. Furthermore, a high level of knowledge may unintentionally foster an expectation of discrimination and the development of stigmatizing attitudes within the community. This unexpected correlation between knowledge and stigma necessitates further investigation and consideration in the discourse on ADHD awareness and societal attitudes. This association is likely influenced by the reliability of the tools used to measure both concepts. Nevertheless, education and advocacy would still be essential to reducing ADHD stigma [47].

Additionally, our results showed that individuals who have received a diagnosis of ADHD exhibited lower levels of stigma related to the disorder, as indicated by their scores on the ASQ scale. Although the current sample included uneven percentages of individuals with and without ADHD (5% vs. 95%, respectively), this substantial difference in percentages was expected owing to the low prevalence of ADHD in Lebanon (3 per 1000) [1]. Previous findings indicated that a reduction in children’s perceived levels of stigma might have a beneficial impact on the child’s acceptance of their disease and treatment adherence [43]. These findings could stem from various factors, including the natural tendency for individuals to be less likely to stigmatize conditions they themselves experience. Additional contributory factors include increased awareness and acceptance of ADHD following diagnosis, access to appropriate treatment and support services, and a sense of validation and understanding of their experiences. Conversely, individuals who have not received a diagnosis may face greater uncertainty, stigma, and misconceptions surrounding ADHD, leading to higher levels of stigma as measured by the ASQ scale. Finally, participants who consumed alcohol showed lower levels of stigma. The theoretical basis for comparing alcohol use and ADHD stigma levels lies in the shared social and psychological mechanisms underpinning stigma. Both conditions are often viewed through lenses of personal responsibility and moral failing, leading to similar stigmatization patterns. This comparison can help uncover broader societal attitudes and inform interventions targeting stigma reduction for both groups [2]. Individuals who consume alcohol may score lower on the ASQ scale due to a better understanding of the stigma experience and social judgment, making them more empathetic and less likely to stigmatize others with different conditions. Additionally, they may perceive ADHD and its behaviors as less controllable and, thus, less blameworthy. This shared perspective on stigmatization can reduce their overall stigma levels toward ADHD [3, 4].

### Public health implications

Our findings indicate that targeted awareness campaigns, including web-based educational initiatives and televised programs, hold promise for enhancing knowledge while also mitigating misconceptions and stigmatizing beliefs surrounding ADHD. Such efforts can foster a more tolerant, patient, and empathetic societal attitude toward ADHD, extending beyond healthcare professionals to the broader community. Furthermore, the results underscore the necessity for inclusive awareness campaigns recognizing the varied impact of ADHD across age groups, countering the widespread misconception that this condition predominantly affects younger male individuals in the Lebanese community. Raising awareness through these initiatives has the potential to combat stigma, rectify misdiagnoses, and instill more informed and nuanced approaches to ADHD diagnosis and care.

### Limitations and strengths

Several limitations could be noted, mainly due to the cross-sectional design of our study. Sampling bias is possible due to the online recruitment strategy, and the reliance on online questionnaires may have introduced bias related to internet literacy. Additionally, the predominantly young, highly educated participant group may not fully represent the broader population, further contributing to potential sampling bias. Also, the translation procedure of the questionnaire did not involve the standard approach of forward and back translation, which could have provided additional validation of the translation’s accuracy and cultural relevance. Nevertheless, our study contributes significantly to the limited knowledge of ADHD in the Middle East, particularly Lebanon. The KADDS tool was not validated in Lebanon or the Arab region; thus, some results, such as the positive correlation between ADHD knowledge and related stigma, require future re-examination of the tool’s validity in the Lebanese context. The use of validated tools would have ensured robust measurements while providing novel insights into ADHD. Caution is also warranted in generalizing findings due to inherent limitations in the study design and participant demographics. Furthermore, although multivariable analyses were applied to decrease confounding, residual confounding bias is still possible due to unmeasured potential confounders. Further studies accounting for these weaknesses are suggested to confirm our findings.

## Conclusions

In summary, this study contributes to the understanding of knowledge and stigma related to ADHD within the general Lebanese population, addressing a notable gap in the existing literature. Demographic correlates, including age, gender, education, and socioeconomic status, highlight the variable impact of public mental health education. An unexpected positive correlation between heightened ADHD knowledge and increased stigma challenges conventional expectations, indicating a complex interplay between knowledge, misconceptions, and societal attitudes. This finding underscores the imperative for targeted education and advocacy efforts to enhance knowledge and alleviate misconceptions and stigmatizing beliefs in the general population.

## Supporting information

S1 Appendix (DOCX)

S1 Table Description of the scales used in the study. (DOCX)

S2 Table A one-way analysis of variance (ANOVA) comparing the association between monthly income and knowledge and stigma toward ADHD. (DOCX)

S1 Fig Normality P-P Plot Graph considering the two dependent variables the KADDS total scale (A) and the ASQ stigma scale (B).
(DOCX)

S2 Fig Scatter plot of the Cook’s distance when considering the KADDS total scale (A) and the ASQ stigma scale (B) as the dependent variables.
(DOCX)
