# Supplementary material for: Exploring ADHD understanding and stigma: Insights from an online survey in Lebanon
Source: PLoS One. 2024 Nov 14;19(11):e0310755. doi: 10.1371/journal.pone.0310755 (PMC11563464; doi:10.1371/journal.pone.0310755)
Supplement: S1 Appendix — (DOCX) [file pone.0310755.s001.docx]

**Knowledge and stigma related to Attention-Deficit/Hyperactivity Disorder (ADHD) among the general Lebanese population**

**المعرفة والوصمة المرتبطة باضطراب نقص الانتباه وفرط النشاط (ADHD) بين عموم السكان اللبنانيين**

Dear Participant,

We are conducting a study to assess the level of knowledge and stigma regarding Attention-Deficit/Hyperactivity Disorder (ADHD) among the general Lebanese population.

If you are Lebanese and over the age of 18, you are invited to participate in this survey, which will take approximately 10 minutes of your time. Please note that all your answers will remain anonymous and confidential and will never be linked to any personally identifiable information. Participation in this survey is voluntary, and your completion of this survey constitutes your written consent to participate.

Thank you for your time and cooperation.

The Research Team

عزيزي المشارك ،

نحن نجري دراسة لتقييم مستوى المعرفة والوصمة فيما يتعلق باضطراب نقص الانتباه / فرط النشاط (ADHD) بين عموم السكان اللبنانيين.

إذا كنت لبنانيًا وتجاوزعمرك 18 عامًا، فأنت مدعو للمشاركة في هذا الاستطلاع، والذي سيستغرق حوالي 10 دقائق من وقتك. يرجى الملاحظة أن جميع إجاباتك ستبقى مجهولة وسرية ولن يتم ربطها بأي معلومات شخصية. المشاركة في هذا الاستطلاع طوعية، ويشكل إكمال هذا الاستطلاع موافقتك الكتابية على المشاركة.

شكرا لك على وقتك وتعاونك.

فريق البحث

**Informed consent.**

*Please check all the boxes to proceed to the survey:*

I have read and understood the above information لقد قرأت وفهمت المعلومات الواردة أعلاه

I understand that my participation is voluntary أدرك أن مشاركتي طوعية

I understand that my data will be kept confidential أدرك أن المعلومات التي سأقدمها ستبقى سرية

I agree to participate in this study أوافق على المشاركة في هذه الدراسة

**Part A. Demographic Data** **الجزء أ. البيانات الديموغرافية**

**A1. Gender الجنس**

Male ذكر

Female أنثى

**A2. Age العمر**

**A3. Area of residence مكان الاقامة**

Beqaa البقاع

Baalbeck/Hermel بعلبك/الهرمل

Mount Lebanon جبل لبنان

Beirut بيروت

Keserwan-Jbeil كسروان-جبيل

North الشمال

South الجنوب

Akkar عكار

Nabatieh النبطية

**A4. Marital Status الحالة الاجتماعية**

Single أعزب

Marriedمتزوج

Divorced/Widowed/Separated مطلق/أرمل/منفصل

**A5. Number of children (Put 0 if none or not applicable) عدد الأطفال (ضع صفر في حال عدم وجود أطفال)**

**A6. Educational level المستوى التعليمي**

Primary إبتدائي

Intermediate متوسط

Secondary ثانوي

University جامعي

Postgraduate دراسات عليا

**A7. Occupation المهنة**

Unemployed عاطل عن العمل حاليا

Student in a health-related field طالب في مجال متعلق بالصحة

Student in a non-health-related field طالب في مجال غير متعلق بالصحة

Working in a health-related jobموظف في مجال متعلق بالصحة

Working in a non-health-related job موظف في مجال غير متعلق بالصحة

Retiredمتقاعد

**A8. Alcohol consumption** **استهلاك الكحول**

Do you drink alcohol? هل تشرب المشروبات الكحولية

Yes, frequently (more than once weekly) نعم ، بشكل متكرر (أكثر من مرة في الأسبوع)

Yes, occasionally (once weekly or less) نعم ، من حين لآخر (مرة أسبوعيًا أو أقل)

No لا

**Smoking التدخين**

**A9.1 Do you smoke cigarettes? هل تدخن السجائر؟**

Yes, current smoker نعم ، مدخن حالي

Yes, previous smoker نعم ، مدخن سابق

No لا

**A.9.2 Do you smoke nargileh?** **هل تدخن النرجيلة؟**

Yes, current smoker نعم ، مدخن حالي

Yes, previous smoker نعم ، مدخن سابق

No لا

**A.9.3 Do you use e-cigarettes/vaping devices?**

**هل تستخدم السجائر الإلكترونية / أجهزة vaping؟**

Yes, currently نعم ، حاليا"

Yes, I used to نعم ، سابقا"

No لا

**A10. Household monthly income الدخل الشهري للأسرة**

< 3,000,000 LBP

3,000,000 – 6,000,000 LBP

6,000,001 – 9,000,000 LBP

9,000,001 – 15,000,000 LBP

15,000,001 – 30,000,000 LBP

> 30,000,000 LBP

Don't know/I prefer not to answer لا أعرف/ أفضل أن لاأجاوب

**A11. Do you have any health coverage? هل لديك أي تأمين صحي؟**

Yes, NSSFالصندوق الوطني للضمان الاجتماعي نعم ،

Yes, COOP نعم ، تعاونية

Yes, army نعم ، جيش

Yes, private insurance نعم ، تأمين خاص

Yes, other، غيره نعم

No لا

**A12. Do you have experience with individuals with ADHD? هل لديك خبرة مع أشخاص لديهم اضطراب نقص الانتباه / فرط النشاط؟**

Yes نعم

No لا

I don't know لا أعلم

**A13. Please indicate the closest approximation of your relationship يرجى تحديد قرب علاقتك**

No experience لا خبرة

Immediate family فرد من أفراد الأسرة

Extended family

Friend or acquaintance صديق أو معارف

Patient of mine مريضي

Other غيره

**A14. Have you ever been diagnosed with ADHD? هل سبق أن تم تشخيصك باضطراب فرط الحركة ونقص الانتباه؟**

Yes نعم

No لا

**A15. How much do you know about ADHD? كم تعرف عن اضطراب فرط الحركة ونقص الانتباه؟**

I don’t know anything about it لا أعرف أي شيء عنه

I have some information about it لدي بعض المعلومات عنه

I have good information about it لدي معلومات جيدة عنه

**A16. Have you ever attended a conference or seminar on ADHD? هل سبق لك أن حضرت مؤتمرًا أو ندوة حول اضطراب فرط الحركة ونقص الانتباه؟**

Yes نعم

No لا

**A17. Have you been trained to interact with individuals with ADHD? هل تم تدريبك على التفاعل مع الأفراد المصابين باضطراب فرط الحركة ونقص الانتباه؟**

Yes نعم

No لا

**A18. What are your sources of information about ADHD? ما هي مصادر المعلومات الخاصة بك حول اضطراب فرط الحركة ونقص الانتباه؟**

Family العائلة

Friends الأصدقاء

Books/journals/newspapers/magazines كتب / مجلات / صحف

Internet إنترنت

Social media وسائل التواصل الاجتماعي

Television التلفاز

Class lectures محاضرات صفية

Webinars/ seminars/ conferences ندوات عبر الإنترنت / ندوات / مؤتمرات

**Part B. Knowledge of Attention Deficit Disorders Scale (KADDS) مقياس المعرفة باضطرابات الانتباه والنشاط الحركي الزائد**


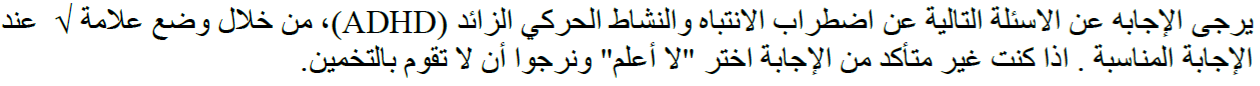


**KADDS ADHD-associated features (15 items)**

1. Children with ADHD are more distinguishable from normal children in a classroom setting than in a free play situation. (TRUE)

يمكن تمييز الأطفال الذين لديهم اضطراب الإنتباه والنشاط الحركي الزائد (ADHD) عن أقرانهم العاديين خلال تواجدهم بالصف الدراسي بشكل أسهل من التمييز بينهم خلال فترة اللعب الحر.

1. Symptoms of ADHD are often seen in non‑ADHD children who come from inadequate and chaotic home environments. (TRUE)

غالباً ما يمكن ملاحظة أعراض الإنتباه والنشاط الحركي الزائد (ADHD) لدى الأطفال الذين ليس لديهم هذا الإضطراب وينتمون إلى بيئة منزلية مضطربة وغير ملائمة.

1. It is possible for an adult to be diagnosed with ADHD. (TRUE)

من الممكن تشخيص الكبار باضطراب الإنتباه والنشاط الحركي الزائد (ADHD).

1. The majority of ADHD children evidence some degree of poor school performance in the elementary school years. (TRUE)

يتغير لدى غالبية الأطفال الذين لديهم اضطراب الإنتباه والنشاط الحركي الزائد (ADHD) من ضعف في الأداء المدرسي خلال المرحلة الابتدائية.

1. If an ADHD child is able to demonstrate sustained attention to video games or TV for over an hour, that child is also able to sustain attention for at least an hour of class or homework. (FALSE)

إذا كان الطفل الذي لديه اضطراب الإنتباه والنشاط الحركي الزائد (ADHD) قادر على الإحتفاظ بإنتباهه لمدة ساعة أو أكثر عند مشاهدة التلفزيون أو ألعاب الفيديو، فأن هذا الطفل يمكنه أيضاً الإحتفاظ بإنتباهه لمدة ساعة على الأقل في الفصل أو عند أداء الواجبات المنزلية.

1. Most ADHD children “outgrow” their symptoms by the onset of puberty and subsequently function normally in adulthood. (FALSE)

يتخلص معظم الأطفال الذين لديهم اضطراب الإنتباه والنشاط الحركي الزائد (ADHD) من أعراض الاضطراب مع بداية مرحلة البلوغ و بالتالي يمارسون حياتهم بشكل طبيعي كغيرهم من البالغين.

1. In school‑age children, the prevalence of ADHD in males and females is equivalent. (FALSE)

تتساوى نسبة انتشار اضطراب الإنتباه والنشاط الحركي الزائد (ADHD) بين الذكور و الإناث من الأطفال في سن المدرسة.

1. Symptoms of depression are found more frequently in ADHD children than in non‑ADHD children. (TRUE)

تبدو أعراض الاكتئاب أكثر شيوعاً لدى الأطفال الذين لديهم اضطراب الإنتباه و النشاط الحركي الزائد (ADHD) عند المقارنة مع غيرهم من الأطفال الذين ليس لديهم هذا الاضطراب.

1. There are specific physical features which can be identified by medical doctors (e.g., pediatrician) in making a definitive diagnosis of ADHD. (FALSE)

هناك بعض المظاهر الجسمية المحددة لاضطراب الإنتباه والنشاط الحركي الزائد (ADHD) التي يمكن للأطباء (كطبيب أطفال) أن يتعرفوا عليها مما يجعلهم متأكدين من تشخيص هذا الاضطراب.

1. ADHD is more common in the first-degree biological relatives (i.e., mother, father) of children with ADHD than in the general population. (TRUE)

ينتشر اضطراب الإنتباه والنشاط الحركي الزائد (ADHD) بنسبة أكبر بين الأقارب من الدرجة الأولى (أي الأم و الأب) للطفل الذي لديه الإضطراب عند المقارنة مع عامة المجتمع.

1. A diagnosis of ADHD by itself makes a child eligible for placement in special education. (FALSE)

تشخيص الطفل بأن لديه اضطراب الإنتباه والنشاط الحركي الزائد (ADHD) بحد ذاته يؤهل الطفل للحصول على خدمات التربية الخاصة للإستفادة منها.

1. In very young children (less than 4-year-old), the problem behaviors of ADHD children (e.g., hyperactivity, inattention) are distinctly different from age‑appropriate behaviors of non‑ADHD children. (FALSE)

الأطفال الصغار (أقل من أربع سنوات) الذين لديهم اضطراب الإنتباه والنشاط الحركي الزائد (ADHD) تختلف مشاكلهم السلوكية (كعدم الإنتباه وفرط الحركة) إختلافاً واضحاً عن السلوكيات المناسبة للأطفال ممن هم في نفس العمر وليس لديهم هذا الاضطراب.

1. ADHD children are typically more compliant with their fathers than with their mothers. (TRUE)

يميل الأطفال الذين لديهم اضطراب الإنتباه والنشاط الحركي الزائد (ADHD) إلى الإنصياع وإطاعة آباءهم بدرجة أكبر من أمهاتهم.

1. Most estimates suggest that ADHD occurs in approximately 15% of school‑age children. (FALSE)

تشير معظم التقديرات إلى أن حوالي 15% من الأطفال في سن المدرسة لديهم اضطراب الإنتباه والنشاط الحركي الزائد (ADHD).

1. ADHD children generally experience more problems in novel situations than in familiar situations. (FALSE)

الأطفال الذين لديهم اضطراب الإنتباه والنشاط الحركي الزائد (ADHD) يواجهون في الغالب مشاكل أكثر في المواقف الجديدة (غير المألوفة) مقارنة بالمواقف المألوفة.

**KADDS Symptoms/Diagnosis (9 items)**

1. ADHD children often fidget or squirm in their seats. (TRUE)

كثيراً ما يقوم الأطفال الذين لديهم اضطراب الإنتباه والنشاط الحركي الزائد (ADHD) بالتململ أثناء جلوسهم بالمقعد.

1. ADHD children often have difficulties organizing tasks and activities. (TRUE)

غالباً ما يواجه الأطفال الذين لديهم اضطراب الإنتباه والنشاط الحركي الزائد (ADHD) صعوبات في تنظيم المهام والأنشطة.

1. ADHD children are frequently distracted by extraneous stimuli. (TRUE)

كثيراً ما تؤدي المثيرات الخارجية (مثل الأصوات خارج الفصل) إلى تشتيت تركيز الأطفال الذين لديهم اضطراب الإنتباه والنشاط الحركي الزائد (ADHD).

1. Current wisdom about ADHD suggests two clusters of symptoms: One of inattention and another consisting of hyperactivity/impulsivity. (TRUE)

حسب الفهم الحالي لاضطراب الإنتباه والنشاط الحركي الزائد (ADHD)، يمكن تصنيف أعراض هذا الاضطراب إلى مجموعتين: الأولى تضم قلة الإنتباه والثانية تشمل فرط الحركة و الإندفاعية.

1. In order to be diagnosed as ADHD, a child must exhibit relevant symptoms in two or more settings (e.g., home, school). (TRUE)

يجب أن تظهر على الطفل أعراض اضطراب الإنتباه والنشاط الحركي الزائد (ADHD) في موضعين أو أكثر (كالمنزل والمدرسة) وذلك قبل أن يتم التشخيص بهذا الاضطراب.

1. One symptom of ADHD children is that they have been physically cruel to other people. (FALSE)

إحدى سمات الأطفال الذين لديهم اضطراب الإنتباه والنشاط الحركي الزائد (ADHD) هي الإيذاء الجسدي للآخرين.

1. It is common for ADHD children to have an inflated sense of self‑esteem or grandiosity. (FALSE)

يشيع بين الأطفال الذين لديهم اضطراب الإنتباه والنشاط الحركي الزائد (ADHD) المبالغة في تقدير الذات الايجابي.

1. ADHD children often have a history of stealing or destroying other people’s things. (FALSE)

غالباً ما يكون لدى الأطفال الذين لديهم اضطراب الإنتباه والنشاط الحركي الزائد (ADHD) تجارب سابقة في السرقة أو تدمير ممتلكات الآخرين.

1. In order to be diagnosed with ADHD, the child’s symptoms must have been present before age 7. (TRUE)

يجب أن تظهر أعراض اضطراب الإنتباه والنشاط الحركي الزائد (ADHD) على الطفل قبل سن السابعة حتى يتم تشخيصه بهذا الاضطراب.

**KADDS Treatment (12 items)**

1. Parent and teacher training in managing an ADHD child are generally effective when combined with medication treatment. (TRUE)

إن تدريب الوالدين والمعلمين للتعامل مع الطفل الذي لديه اضطراب الإنتباه والنشاط الحركي الزائد (ADHD) يكون أكثر فعالية إذا تزامن مع استخدام العلاج الطبي.

1. Individual psychotherapy is usually sufficient for the treatment of most ADHD children. (FALSE)

العلاج النفسي الفردي عادة يكون كافي لمعالجة معظم الأطفال الذين لديهم اضطراب الإنتباه والنشاط الحركي الزائد (ADHD).

1. Treatments for ADHD which focus primarily on punishment have been found to be the most effective in reducing the symptoms of ADHD. (FALSE)

أكثر أساليب العلاج فاعلية في الحد من أعراض اضطراب الإنتباه والنشاط الحركي الزائد (ADHD) هي التي تركز بشكل رئيسي على العقاب.

1. In severe cases of ADHD, medication is often used before other behavior modification techniques are attempted. (TRUE)

يستخدم العلاج الدوائي كثيراً في الحالات الشديدة من اضطراب الإنتباه والنشاط الحركي الزائد (ADHD) قبل البدء باستخدام فنيات تعديل السلوك.

1. When treatment of an ADHD child is terminated, it is rare for the child’s symptoms to return. (FALSE)

عند إيقاف علاج الطفل الذي لديه اضطراب الإنتباه والنشاط الحركي الزائد (ADHD) فأنه من النادر عودة أعراض الاضطراب لديه مرة أخرى.

1. Side effects of stimulant drugs used for treatment of ADHD may include mild insomnia and appetite reduction. (TRUE)

يعد الأرق وفقدان الشهية من بين الآثار الجانبية للعقاقير المنشطة المستخدمة لعلاج اضطراب الإنتباه والنشاط الحركي الزائد (ADHD).

1. Antidepressant drugs have been effective in reducing symptoms for many ADHD children. (TRUE)

لقد أثبتت العقاقير المضادة للاكتئاب فعاليتها في الحد من أعراض اضطراب الإنتباه والنشاط الحركي الزائد (ADHD).

1. Electroconvulsive therapy (i.e., shock treatment) has been found to be an effective treatment for severe cases of ADHD. (FALSE)

يعد العلاج الكهربائي (أي المعالجة بالصدمات الكهربائية) فاعلاً في علاج الحالات الشديدة من اضطراب الإنتباه والنشاط الحركي الزائد (ADHD).

1. Current research suggests that ADHD is largely the result of ineffective parenting skills. (FALSE)

الدراسات الحالية تشير إلى أن اضطراب الإنتباه والنشاط الحركي الزائد (ADHD) في الغالب يكون نتيجة لتدني مهارات لوالدين في تربية وتنشئة الطفل.

1. Behavioral/Psychological interventions for children with ADHD focus primarily on the child’s problems with inattention. (FALSE)

يركز التدخل العلاجي السلوكي/النفسي للأطفال الذين لديهم اضطراب الإنتباه والنشاط الحركي الزائد (ADHD) في المقام الأول على مشاكل قلة الإنتباه لدى الطفل.

1. Stimulant drugs are the most common type of drug used to treat children with ADHD. (TRUE)

العقاقير المنشطة هي أكثر الأدوية استخداماً لعلاج حالات اضطراب الإنتباه والنشاط الحركي الزائد (ADHD).

1. Reducing dietary intake of sugar or food additives is generally effective in reducing the symptoms of ADHD. (FALSE)

خفض كمية ما يتم تناوله من السكريات والمواد الحافظة يساعد في الحد من أعراض اضطراب الإنتباه والنشاط الحركي الزائد (ADHD).

**Part C. Attention-Deficit/Hyperactivity Disorder (ADHD) Stigma Questionnaire**

*This set of questions asks about some of the experiences, feelings, and opinions people with ADHD might have and how they are treated. Please do your best to answer each question. For each item, choose your answer: Strongly disagree (SD), Disagree (D), Neutral (N), Agree (A), or Strongly agree (SA).*

*تسأل هذه المجموعة من الأسئلة عن بعض التجارب والمشاعر والآراء التي قد يمتلكها الأشخاص المصابون باضطراب فرط الحركة ونقص الانتباه وكيفية معاملتهم. الرجاء بذل قصارى جهدك للإجابة على كل سؤال. لكل عنصر ، اختر إجابتك: لا أوافق بشدة (SD) ، لا أوافق (D) ، محايد (N) ، أوافق (A) ، أو أوافق بشدة (SA).*

1. People who have ADHD feel guilty about it. يشعر الأشخاص المصابون باضطراب فرط الحركة ونقص الانتباه بالذنب حيال ذلك.
2. People’s attitudes about ADHD may make persons with ADHD feel worse about themselves. قد تجعل مواقف الناس حول اضطراب فرط الحركة ونقص الانتباه الأشخاص المصابين به يشعرون بسوء تجاه أنفسهم.
3. Someone who has ADHD would think it’s risky to tell others about it. قد يعتقد الشخص المصاب باضطراب فرط الحركة ونقص الانتباه أنه من الخطر إخبار الآخرين عنه.
4. People with ADHD lose their jobs when their employers find out. الأشخاص المصابون باضطراب فرط الحركة ونقص الانتباه يفقدون وظائفهم عندما يكتشف أصحاب العمل ذلك.
5. People with ADHD work hard to keep it a secret. الأشخاص المصابون باضطراب فرط الحركة ونقص الانتباه يعملون بجد لإبقائه سراً.
6. Someone with ADHD feel they aren’t as good a person as others because they have ADHD. يشعر الشخص المصاب باضطراب فرط الحركة ونقص الانتباه بأنه ليس جيدًا مثل الآخرين لأنه مصاب بهذا المرض.
7. People with ADHD are treated like outcasts. يُعامل الأشخاص المصابون باضطراب فرط الحركة ونقص الانتباه مثل المنبوذين.
8. People with ADHD feel damaged because of it. يشعر الأشخاص المصابون باضطراب فرط الحركة ونقص الانتباه بالضرر بسببه.
9. After learning they have ADHD, a person may feel set apart and isolated from the rest of the world. بعد معرفة أنه مصاب باضطراب فرط الحركة ونقص الانتباه ، قد يشعر الشخص بأنه منفصل ومنعزل عن بقية العالم.
10. Most people think that a person with ADHD is damaged. يعتقد معظم الناس أن الشخص المصاب باضطراب فرط الحركة ونقص الانتباه قد تضرر.
11. A person with ADHD feels that they are bad because of it. يشعر الشخص المصاب باضطراب فرط الحركة ونقص الانتباه أنه سيء ​​بسببه.
12. Most people with ADHD are rejected when others find out. يتم رفض معظم الأشخاص المصابين باضطراب فرط الحركة ونقص الانتباه عندما يكتشف الآخرون ذلك.
13. People who have ADHD are very careful about who they tell. الأشخاص الذين يعانون من اضطراب فرط الحركة ونقص الانتباه حريصون جدًا على من يخبرون.
14. Some people who learn of another person having ADHD grow distant. بعض الناس الذين يكتشفون ان شخصا" آخرا" مصابا" باضطراب فرط الحركة ونقص الانتباه يبعدون عنه.
15. After learning they have ADHD, people worry about others discriminating against them. بعد أن يكتشف الناس أنهم مصابون باضطراب فرط الحركة ونقص الانتباه ، يقلقون بشأن تمييز الآخرين ضدهم.
16. Most people are uncomfortable around someone with ADHD. معظم الناس غير مرتاحين حول شخص مصاب باضطراب فرط الحركة ونقص الانتباه.
17. People with ADHD worry that others may judge them when they learn that they have ADHD. يشعر الأشخاص المصابون باضطراب فرط الحركة ونقص الانتباه بالقلق من أن الآخرين قد يحكمون عليهم عندما يعلمون أنهم مصابون بهذا المرض.
18. People with ADHD regret having told some people that they have ADHD. الأشخاص المصابون باضطراب فرط الحركة ونقص الانتباه يندمون على إخبار بعض الأشخاص بأنهم مصابون بهذا المرض.
19. As a rule, People with ADHD feel that telling others that they have ADHD was a mistake. كقاعدة عامة ، يشعر الأشخاص المصابون باضطراب فرط الحركة ونقص الانتباه أن إخبار الآخرين بأنهم يعانون من اضطراب نقص الانتباه مع فرط النشاط كان خطأ.
20. People don’t want someone with ADHD around their children once they know that person has ADHD. لا يرغب الناس في وجود شخص مصاب باضطراب فرط الحركة ونقص الانتباه حول أطفالهم بمجرد أن يعرفوا ذلك الشخص
21. Some people act as though it’s the person’s fault that they have ADHD. يتصرف بعض الأشخاص كما لو أنه خطأ الشخص أن لديهم اضطراب فرط الحركة ونقص الانتباه.
22. People with ADHD have lost friends by telling them they have ADHD. الأشخاص المصابون باضطراب فرط الحركة ونقص الانتباه فقدوا أصدقاء بإخبارهم أنهم مصابون بهذا المرض.
23. People with ADHD have told others close to them to keep the fact that they have ADHD a secret. الأشخاص المصابون باضطراب فرط الحركة ونقص الانتباه أخبروا الآخرين المقربين منهم أن يحافظوا على حقيقة أنهم مصابون باضطراب فرط الحركة ونقص الانتباه.
24. The good points of people with ADHD tend to be ignored. النقاط الجيدة لدى الأشخاص المصابين باضطراب فرط الحركة ونقص الانتباه مائلة إلى تجاهل.
25. People seem afraid of a person with ADHD once they learn they have ADHD. يبدو أن الناس يخافون من الشخص المصاب باضطراب فرط الحركة ونقص الانتباه بمجرد أن يعلموا أنه مصاب بهذا المرض.
26. When people learn that someone has ADHD, they look for flaws in their character. عندما يعلم الناس أن شخصًا ما مصاب باضطراب فرط الحركة ونقص الانتباه ، فإنهم يبحثون عن عيوب في شخصيتهم.
