# Supplementary material for: Exploring ADHD understanding and stigma: Insights from an online survey in Lebanon
Source: PLoS One. 2024 Nov 14;19(11):e0310755. doi: 10.1371/journal.pone.0310755 (PMC11563464; doi:10.1371/journal.pone.0310755)
Supplement: S1 Table — (DOCX) [file pone.0310755.s002.docx]

**S2 table 1: Description of the scales used in the study**

| **Supplementary table 1: Description of the scales used in the study** | | | | | | |
| --- | --- | --- | --- | --- | --- | --- |
|  | **Median** | **Mean** | **Mean %** | **SD** | **Minimum** | **Maximum** |
| **Knowledge of Attention Deficit Disorders Scale (KADDS)** | 20.00 | 20.49 | 56.91 | 3.23 | 10.00 | 34.00 |
| KADDS ADHD-associated features | 8.00 | 8.03 | 53.53 | 1.65 | 3.00 | 15.00 |
| KADDS Symptoms/Diagnosis | 6.00 | 5.53 | 61.44 | 1.92 | 1.00 | 9.00 |
| KADDS Treatment | 7.00 | 6.92 | 57.66 | 1.56 | 2.00 | 12.00 |
| **Attention-Deficit/Hyperactivity Disorder (ADHD) Stigma Questionnaire** | 78.00 | 75.71 | 58.23 | 20.58 | 26.00 | 130.00 |
