# Supplementary material for: Exploring ADHD understanding and stigma: Insights from an online survey in Lebanon
Source: PLoS One. 2024 Nov 14;19(11):e0310755. doi: 10.1371/journal.pone.0310755 (PMC11563464; doi:10.1371/journal.pone.0310755)
Supplement: S2 Table — (DOCX) [file pone.0310755.s003.docx]

**S3 table 2: A one-way analysis of variance (ANOVA) comparing the association between monthly income and knowledge and stigma toward ADHD**

| **Supplementary table 2: A one-way analysis of variance (ANOVA) comparing the association between monthly income and knowledge and stigma toward ADHD** | | | | | | | |
| --- | --- | --- | --- | --- | --- | --- | --- |
|  | Total N | Knowledge of Attention Deficit Disorders Scale (KADDS) | **F-value** | **p-value*** | Attention-Deficit/Hyperactivity Disorder (ADHD) Stigma Questionnaire | **F-value** | **p-value*** |
| **Monthly income#** |  |  |  |  |  |  |  |
| Don’t know | 242 | 20.47±3.10 | 6.18 | **<0.001** | 78.03±17.72 | 5.37 | **0.001** |
| Low (<100 USD) | 114 | 20.23±2.80 |  |  | 73.15±23.41 |  |  |
| Intermediate  (100-335 USD) | 152 | 19.86±3.03 |  |  | 71.06±22.40 |  |  |
| High (>335 USD) | 139 | 21.43±3.75 |  |  | 78.84±19.71 |  |  |
| *Number in bold are statistically significant (< 0.05).  # Post-hoc analysis of the association between the means of KADDS scale and monthly income: don’t know vs high p=0.030; don’t know vs intermediate p=0.401; don’t know vs low p=1.000; high vs intermediate p=<0.001; high vs low p=0.019; intermediate vs low p=1.000.  Post-hoc analysis of the association between the means of stigma scale and monthly income: don’t know vs high p=1.000; don’t know vs intermediate p=0.006; don’t know vs low p=0.214; high vs intermediate p=0.007; high vs low p=0.166; intermediate vs low p=1.000. | | | | | | | |
