# Supplementary material for: Exploring ADHD understanding and stigma: Insights from an online survey in Lebanon
Source: PLoS One. 2024 Nov 14;19(11):e0310755. doi: 10.1371/journal.pone.0310755 (PMC11563464; doi:10.1371/journal.pone.0310755)
Supplement: S1 Fig — Normality P-P Plot Graph considering the two dependent variables the KADDS total scale (A) and the ASQ stigma scale (B). (DOCX) [file pone.0310755.s004.docx]

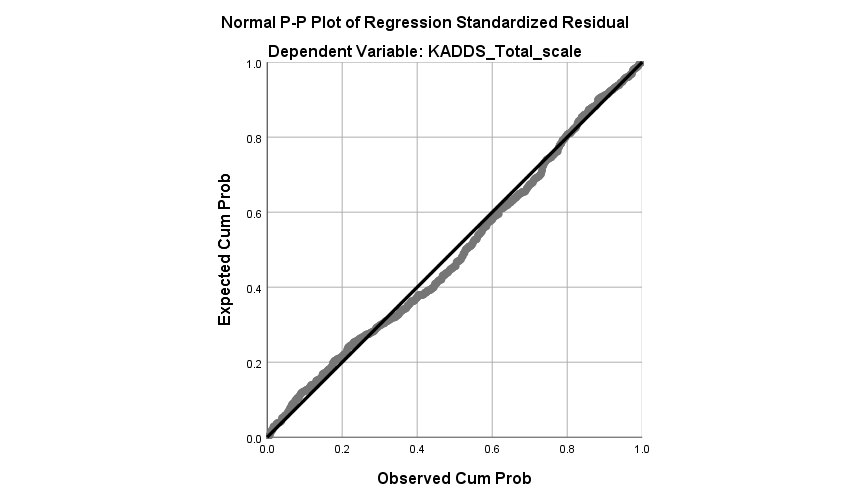

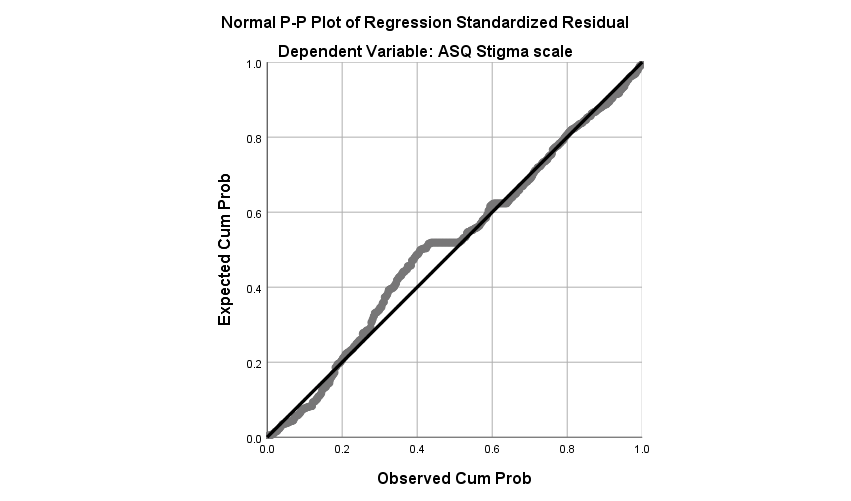


B

A

**S4 figure 1: Normality P-P Plot Graph considering the two dependent variables the KADDS total scale (A) and the ASQ stigma scale (B)**
